# Validation of a New Larval Rearing Unit for *Aedes albopictus* (Diptera: Culicidae) Mass Rearing

**DOI:** 10.1371/journal.pone.0091914

**Published:** 2014-03-19

**Authors:** Fabrizio Balestrino, Arianna Puggioli, Jérémie R. L. Gilles, Romeo Bellini

**Affiliations:** 1 Insect Pest Control Laboratory, Joint Food and Agriculture Organization/International Atomic Energy Agency (FAO/IAEA) Division of Nuclear Techniques in Food and Agriculture, International Atomic Energy Agency (IAEA), Vienna, Austria; 2 Medical and Veterinary Entomology Department, Centro Agricoltura Ambiente C.A.A. “G. Nicoli”, Crevalcore, Italy; Centro de Pesquisas René Rachou, Brazil

## Abstract

The mosquito larval rearing unit developed at the Insect Pest Control Laboratory (IPCL) of the FAO/IAEA Joint Division was evaluated for its potential use for *Aedes albopictus* (Skuse, 1895) mass rearing in support of the development of a sterile insect technique (SIT) package for this species. The use of the mass rearing trays and rack did not adversely affect larval development, pupation and survival rates and allowed the management of large larval rearing colonies with reduced space requirements in comparison with classical individual trays. The effects of larval density, water temperature and diet composition on pupal production and size differentiation for sex separation efficacy were analyzed for individual mass rearing trays as well as multiple trays stacked within the dedicated rack unit. Best results were obtained using eighteen thousand larvae per tray at a density of 3 larvae per ml of deionized water at a temperature of 28°C on a diet consisting of 50% tuna meal, 36% bovine liver powder, 14% brewer's yeast and, as an additive, 0.2 gr of Vitamin Mix per 100 ml of diet solution. Pupae were harvested on the sixth day from larval introduction at L_1_ stage and males were separated out by the use of a 1400 µm sieve with 99.0% accuracy with a recovery rate of ca. 25% of the total available males. With the use of this larval rearing unit, an average production of 100,000 male pupae per week can be achieved in just 2 square meter of laboratory space. Compared to previous laboratory rearing method, the same pupal production and sex separation efficacy could only be achieved by use of ca. 200 plastic trays which required the space of two 5 square meter climatic-controlled rooms.

## Introduction

Over the last decades, vector-borne diseases have emerged and resurged affecting nearly half of the world's population and resulting in high morbidity and mortality [1]. Despite ongoing control efforts which are mainly based on the use of insecticides and elimination of mosquito larval breeding sites, diseases transmitted by mosquitoes such as malaria and dengue, continue to pose an enormous global public health threat and demands for improved tools and strategies for mosquito control and disease prevention are increasing [2].

The sterile insect technique (SIT) represents one of the first genetic control method applied on a large scale which has achieved considerable success in insect pest eradication or suppression [3], [4]. In its classical approach, where irradiation is used to sexually sterilize males, the SIT has been applied on several species of medical, veterinary and agronomic importance over the past 60 years [5]. Despite several trials performed during the 1970's and 80's which showed the possibility to successfully reduce natural mosquito populations, no further efforts have been made in order to move this technology toward larger scale control trials using radiation-based SIT [6]. In parallel, different approaches employing genetically modified or Wolbachia-infected mosquitoes have been proposed as a possible tool for the control of mosquitoes and mosquito-borne diseases [6], [7]. Regardless of the different approaches, these genetic control methods share some significant similarities like the extreme species-specificity with minimal impact on non-target organisms and the area-wide approach accomplished by measures whose effectiveness depends on application over large areas initially or continuously supported by efficient mass production of modified target pest [8]. The major obstacles for the development of SIT and/or other methodologies for the control of mosquitoes at large scale include the lack of efficient rearing methods in order to consistently produce a large number of high quality males, the ability to reliably sort males from females, the availability of effective methods to sterilize the males and an efficient mechanism to distribute the sterile males produced. In the advent of new technologies, a wide variety of tools have been made available providing solutions to these issues, therewith renewing interest in the application of the genetic methods for area-wide mosquito control programs. In order to support the development of area-wide mosquito control measures with an SIT component, the FAO/IAEA Insect Pest Control Laboratory (IPCL) is developing, among several other techniques, methods and technologies for large-scale, simple and mechanized mosquito mass rearing procedures [9], [10], [11].

The Asian tiger mosquito, *Aedes albopictus* (Skuse, 1895), is one of the most invasive species in the world and, in addition to its diurnal biting behaviour, was the main vector in recent epidemics of chikungunya (CHIKV) and dengue (DENV) viruses in temperate and tropical areas [12]. Preliminary studies and pilot release programs of *Ae. albopictus* positively confirmed the feasibility of the use of radiation-based SIT against this species as an effective tool to suppress the natural population [13], [14], [15].

The maximal exploitation of the larval mass rearing potential requires making use of the highest possible larval density at which a complete, fast and homogenous development can be assured whilst restoring adult male competitiveness. However, the density at which the larvae can be reared successfully is not an independent parameter but depends on several factors including water temperature [16], [17], [18], water depth [19], [20], [21], food quality and quantity [17], [22], the interaction of which has not been uniquely identified. A strong negative correlation between larval density and the percentage of survival, larval stage duration, pupal and adult size has often been reported although larval nutrition instead of space requirement or intraspecific competition seems to be the most important limiting factor for Aedinae larval development [21], [23]. In respect to *Ae. albopictus*, preliminary studies showed that larval densities between 0.5 and 5.0 larvae per ml do not affect survival until pupal and adult stage, pupation or emergence rate when appropriate larval nutrition is provided (Puggioli unpublished data).

Shallow breeding sites can be considered the most appropriate environment for a successful larval development while increased water depth has been reported to negatively affect the pupation success on several mosquitoes [20], [21]. The use of artificial rearing environments with reduced water depth facilitates larval development, probably by limiting the energy loss through foraging and diving response to alarm stimuli [24], [25]. The stimuli which trigger defensive immersion may include mechanical disturbances, water vibrations or changes in light intensity [24], [26], [27]. The immature stages of *Ae. albopictus*, similarly to *Aedes aegypti* (L.), escape the light and aggregate in the darkest region of trays showing a pronounced negative phototactic reaction [28].

The FAO/IAEA larval rearing unit consists of a mechanized stainless steel rack that can hold 50 large mosquito mass-rearing trays [10]. Due to its shape and its reduced illumination when stacked within the rack, the tray appears appropriate for *Ae. albopictus* mass rearing procedures. In the present study, the evaluation tests and the settings obtained with the use of the FAO/IAEA larval rearing unit on *Ae. albopictus* are reported with an emphasis on the effect of larval density, water temperature and diet composition on larval survival, pupal productivity and sex separation efficacy on *Ae. albopictus*.

## Materials and Methods

### Ethics Statement

Research carried out on invertebrates such as mosquitoes do not require a specific permit according to the directive 2010/63/EU of the European Parliament and of the Council on the protection of animals used for scientific purposes. The experiments were performed inside the Biosafety Level 3 laboratory - BL3 (Ministry of Health, Italian Ministerial Legislative Decree, 626/94, Annex XII) of the Medical and Veterinary Entomology Department, Centro Agricoltura Ambiente “G. Nicoli”- IAEA collaborating Centre in Crevalcore, Italy, in respect to the Standard Operating Procedure in force in a mosquito laboratory. The blood used for routine blood-feeding was collected in Camposanto, Italy during routine slaughtering of pigs in a national authorized abattoir (Az. Agr. All. Suini Rubizzani CE IT N2L7D) at the highest possible standards strictly following EU laws and regulations.

### Experiment 1. Effect of larval density on *Ae. albopictus* larval survival, pupal production and sex separation efficacy

The feasibility of larval mass-rearing for *Ae. albopictus* in the new FAO/IAEA mosquito mass-rearing tray (hereafter referred to as tray) has been evaluated at different larval densities. To simulate conditions as presented when trays are stacked closely together in their rack unit [10], the trays were placed on shelves inside a climatic-controlled room (30±0.5°C, 80±5%RH, 14∶10 h L∶D photoperiod) and covered with trays elevated 3 cm over them using plastic spacers. The trays were filled with 6 liters of deionized water and tested at a density of 2, 3 and 4 larvae per ml of water by adding 12000, 18000 and 24000 first instar larvae (L_1_) per tray respectively. The number of larvae for each tray was estimated by hatching eggs after computerized egg paper scanning and counting [13]. Three replicates were performed for each larval density. The water temperature in the trays was measured daily during the experiment using a digital thermometer with a metal water probe (Ama-digit ad 15 th, Buddeberg GmbH, Mannheim, Germany; range −40 to +120°C, resolution 0.1°C). The water level in each tray was recorded in order to observe water lost by evaporation.

IAEA liquid diet, composed of 50% bovine liver powder, 50% tuna meal and 0.2 gr of Vitamin Mix per 100 ml of diet solution as an additive [22] was provided daily to the larvae. For each larval density, different diet concentrations were given in order to provide the same daily dose of food per larva. Three liquid diet solutions at concentrations of 5, 7.5 and 10% (w/v) were prepared and used in the trays with larval densities of 2, 3 and 4 larvae/ml respectively. During the first four days of the experiment, a volume of 50, 100, 150 and 200 ml of diet per day was administered to the larvae, equal to 0.2, 0.4, 0.6 and 0.8 mg/larva/day respectively.

Twenty-four hours after the appearance of the first pupae, all pupae present in each tray (NP) were collected, counted and sexed using a metal sieve with a 1400 µm square-holed mesh [13]. The pupae which passed through the mesh after 3 minutes of sieving (NPP) and those that did not (N PNP) were counted and placed in plastic cups (10×10×5 cm) filled with 200 ml of deionized water and covered with a net for emergence. Twenty-four hours after emergence, adults were killed by freezing at −20°C and then sexed. Pupae were also collected at 48 and 72 hours after the onset of pupation, counted and sexed as described above but were not processed by mechanical sexing. The number of larvae that had not pupated within 72 hours was recorded for each treatment before the test was terminated in order to compare larval survival between larval density treatments. The pupal production, calculated as the number of pupae passed through the mesh in relation to the initial number of first instar larvae used (NPP/NL_1_), was compared between larval density treatments.

### Experiment 2. Effect of brewer's yeast on *Ae. albopictus* pupal production and sex separation efficacy

Previous experiments have shown that the integration of brewer's yeast in the larval diet significantly enhanced the pupal production at 24 h from pupation onset [29], [30]. In order to test the effect of brewer's yeast in a mass rearing setting, a new liquid diet composition has been prepared and tested. This new diet (hereafter called IAEA-BY) was composed of 50% tuna meal, 36% bovine liver powder, 14% brewer's yeast (YBD-1KG, Sigma-Aldrich, St. Louis, MO) and 0.2 gr of Vitamin Mix per 100 ml of diet solution as an additive.

During a preliminary test (EXP 2A) the larval rearing parameters at a density of 2 and 3 larvae/ml using IAEA-BY diet was evaluated. Five replicates were performed for each treatment. Liquid diets at a concentration of 5% and 7.5% (w/v) were prepared and used with larval density equal to 2 and 3 larvae/ml respectively.

A second experiment (EXP 2B) compared the effect of IAEA and IAEA-BY diets on larval developmental parameters. The two liquid diets were prepared at a concentration of 7.5% (w/v) and administered to trays maintained at a larval density of 3 larvae/ml. Three replicates were performed for each diet tested.

Following the protocol described above, we collected and sexed only the pupae produced up to 24 h from the beginning of pupation for both tests. The number of pupae passed through the sieves (NPP), the percentage of males obtained from the pupae sieved (%MPP) and the pupal production (NPP/NL_1_) were evaluated and compared between treatments according to larval density and larval diet tested.

### Experiment 3. Effect of water temperature on *Ae. albopictus* pupal production and sex separation efficacy

To evaluate the effect of water temperature on larval development of *Ae. albopictus*, further tests were carried out. Larvae were kept at a density of 3 larvae/ml and were fed using IAEA-BY diet with the same feeding schedule as described above. Larval rearing parameters were checked at water temperatures of 25.5, 26.5, 28.5 and 29°C, kept constant by adjusting the rearing room's climate control. The water temperature inside the trays was verified daily using a thermometer with a metal probe.

As described in the previous experiments, we collected and sexed only the pupae produced up to 24 h from the beginning of pupation, assessing the number of pupae which passed (NPP) through the sieves, the percentage of males observed in the pupae passed (%MPP) and the pupal production (NPP/NL_1_).

### Experiment 4. Effect of larval tray settings on *Ae. albopictus* pupal production and sex separation efficacy

In order to evaluate the effect of the IAEA rack on *Ae. albopictus* larval development, comparison tests were conducted using ten covered isolated (individually placed) trays and ten trays stacked inside the rack. In both settings, larval density was kept at 3 larvae/ml, IAEA-BY liquid diet (7.5% w/v) was administered as described above and the water temperature was set at 29°C. Because of the effect of the two settings on the trays' water temperature [10], tests were conducted in two different climatic-controlled rooms set at 31°C for isolated trays and at 32°C for trays stacked in the rack. Water temperature in the two tray settings was measured daily by using a thermometer with metal probe.

At 24 h from the beginning of pupation, pupae were collected and sexed, analyzing the same parameters described in the previous test according to the two settings employed. Three replicates of this test were performed. While pupae from each isolated tray were collected, processed and analyzed separately (N = 10 per replicate), pupae from the ten trays stacked in the rack were collected and processed simultaneously therefore one single data was obtained for each replicate (N = 3). In this setting the pupae collected were divided volumetrically in 10 samples of approximately the same size for mechanical sex separation.

### Experiment 5. Effect of water temperature on *Ae. albopictus* pupal production and sex separation efficacy using the rack

In this test, we compared the effect of rearing water temperature of 28°C and 29°C on *Ae.albopictus* larval development using ten trays stacked in the rack. The test was conducted using a larval density of 3 larvae/ml and IAEA-BY liquid diet was administered as described above. We collected, processed and sexed the pupae produced up to 24 h from the beginning of pupation, analyzing the same parameters described in the previous test as a function of the two temperatures. Six and three replicates were conducted respectively at a temperature of 28°C and 29°C.

### Statistical Analysis

All statistical analyses were performed using MiniTab (MiniTab Inc., State College, (PA). The number of pupae produced and collected at 24, 48 and 72 h from the onset of pupation, the number of pupae passed through and retained by the sieves, the percentage of males observed in the pupae collected (%MPP), the larval survival and the number of pupae separated in relation to the initial number of L_1_ used were all evaluated by a generalized linear model (GLM) with Tukey's post-hoc pair wise comparisons and compared between larval density, larval diet or water temperature treatments. Data expressed as a percentage or as a ratio were analyzed with GLM after angular (arcsinsqrt) transformation.

## Results

### Experiment 1. Effect of larval density on *Ae. albopictus* larval survival, pupal production and sex separation efficacy

The pupae produced up to 24 and 72 h from pupation onset (NP) did not vary significantly with larval density (F_(2,6)_ = 4.41, P>0.05 and F_(2,6)_ = 1.66, P>0.05 respectively; Table 1, EXP 1). The pupae produced at 48 h from the beginning of pupation (NP) differ significantly according to larval density (F_(2,6)_ = 5.59, P<0.05) with less pupae produced as compared to 4 larvae/ml (T = 3.37, P<0.05); no difference was observed in the number of pupae produced at 48 h from treatments with densities of 2 and 3 larvae/ml (T = 1.87, P>0.05) or between larval densities of 3 and 4 larvae/ml (T = 1.47, P>0.05; Table 1).

**Table 1:** Pupal production and larval survival rate registered within three days from the beginning of pupation (24, 48 and 72 hr) resulting from varying larval densities.

						24 h				48 h				72 h					
EXP	SET	T°C	DIET	L/ML	N	NP		%M		NP		%M		NP		%M		LARVAL SURVIVAL	
**1**	TRAY	28	IAEA	**2**	3	6614	**a**	71.16	**a**	2576	**a**	32.67	**a**	1757	**a**	9.50	**a**	98.47	**a**
						(1003)		(5.37)		(536)		(11.62)		(585)		(3.38)		(1.16)	
	TRAY	28	IAEA	**3**	3	7531	**a**	78.22	**a**	4322	**ab**	31.83	**a**	3675	**a**	12.18	**a**	96.96	**a**
						(1121)		(4.24)		(343)		(15.46)		(1161)		(4.53)		(1.70)	
	TRAY	28	IAEA	**4**	3	11627	**a**	62.88	**a**	5701	**b**	28.24	**a**	4198	**a**	9.97	**a**	97.78	**a**
						(1607)		(6.83)		(954)		(11.23)		(1135)		(2.95)		(1.99)	

Different letters represent statistically significant difference among means with P<0.05 level (GLM with Tukey's post-hoc pairwise comparisons). Parentheses enclose standard errors of each mean value. EXP = experiment number; SET = rearing method; T°C = water temperature; DIET = larval diet; L/ML = larval density; N = replicates number. NP = pupae produced; %M = percentage of males in the pupal collection; LARVAL SURVIVAL = larval survival rate at 72 h from pupation onset.

The percentage of males observed in the pupae collected at 24, 48 and 72 h from pupation onset (%M) did not vary significantly with the larval density (F_(2,6)_ = 1.81, P>0.05; F_(2,6)_ = 0.03, P>0.05 and F_(2,6)_ = 0.11, P>0.05, respectively; Table 1, EXP 1). The larval survival did not show statistical difference between the larval densities employed (F_(2,6)_ = 0.35, P>0.05; Table 1, EXP 1). The number of pupae collected at 24 h from pupation onset which passed through the sieves (NPP) and their percentage of male (%MPP) did not vary with larval density employed (F_(2,6)_ = 4.96, P>0.05 and F_(2,6)_ = 1.81, P>0.05 respectively; Table 2, EXP 1).

**Table 2:** Pupal production and corresponding sex separation data at 24(SET), water temperature (T°C), larval diet (DIET) and larval densities (L/ML).

EXP	SET	T°C	DIET	L/ML	N	NP		NPP		%MPP		NPP/NL_1_	
**1**	TRAY	28	IAEA	**2**	3	6614	**a**	2361	**a**	97.74	**a**	19.67	**a**
						(1003)		(109)		(1.01)		(0.91)	
	TRAY	28	IAEA	**3**	3	7531	**a**	2985	**a**	97.68	**a**	16.58	**a**
						(1121)		(170)		(0.16)		(0.94)	
	TRAY	28	IAEA	**4**	3	11627	**a**	3004	**a**	97.89	**a**	12.55	**b**
						(1607)		(201)		(0.77)		(0.84)	
**2A**	TRAY	28	IAEA - BY	**2**	5			1440	**a**	99.18	**a**	12.00	**a**
								(160)		(0.34)		(1.33)	
	TRAY	28	IAEA - BY	**3**	5			2960	**b**	0.987	**a**	16.44	**b**
								(227)		(0.40)		(1.26)	
**2B**	TRAY	28	**IAEA**	3	3	7350	**a**	2284	**a**	96.76	**a**	12.69	**a**
						(76)		(173)		(0.30)		(0.96)	
	TRAY	28	**IAEA - BY**	3	3	6850	**a**	1800	**a**	0.990	**b**	10.01	**a**
						(226)		(231)		(0.219)		(1.27)	
**3**	TRAY	**25.5**	IAEA - BY	3	5			3225	**a**	97.66	**ab**	17.91	**a**
								(534)		(0.55)		(2.97)	
	TRAY	**26.5**	IAEA - BY	3	5			3000	**a**	96.50	**a**	16.67	**ab**
								(228)		(0.88)		(1.27)	
	TRAY	**28.5**	IAEA - BY	3	20			1970	**b**	98.75	**b**	11.94	**b**
								(167)		(0.28)		(0.87)	
	TRAY	**29**	IAEA - BY	3	8			2050	**b**	98.13	**ab**	11.39	**ab**
								(108)		(0.32)		(0.60)	
**4**	**TRAY**	**29**	IAEA - BY	3	30			1993	**a**	98.57	**a**	11.78	**a**
								(118)		(0.218)		(0.64)	
	**RACK**	**29**	IAEA - BY	3	3*			1930	**a**	97.52	**a**	10.72	**a**
								(450)		(0.33)		(2.50)	
**5**	**RACK**	**28**	IAEA - BY	3	6*			2252	**a**	98.97	**a**	12.51	**a**
								(166)		(0.12)		(0.92)	
	**RACK**	**29**	IAEA - BY	3	3*			1930	**a**	97.52	**b**	10.72	**a**
								(450)		(0.33)		(2.50)	

Different letters within each column represent statistically significant differences between means with P<0.05 level (GLM with Tukey's post-hoc pairwise comparisons). Parentheses enclose standard errors of each mean value. EXP = Experiment number; N = Replicates number; NPP = pupae passed through the sieve for sex separation; %MPP = percentage of male in the pupae passed; NPP/NL_1_ = pupal production; NPNP = pupae not passed through the sieve for sex separation; %MPNP = percentage of male in the pupae not passed.

*Data from each replicate were generated by processing pupae collected simultaneously from 10 trays stacked in the rack.

The pupal production (NPP/NL_1_) differed significantly between larval density treatments (F_(2,6)_ = 15.85, P<0.01; Table 2, EXP 1). At 4 larvae/ml this parameter was statistically lower in comparison with the value observed at 2 larvae/ml (T = −5.601, P<0.01) and 3 larvae/ml (T = −3.305, P<0.05). No difference was observed between larval density of 2 and 3 larvae/ml (T = −2.296, P>0.05). No significant difference was found in the number of pupae which did not pass through the sieve (NPNP) and in the percentage of males in this fraction between larval density treatments (F_(2,6)_ = 4.48, P<0.05 and F_(2,6)_ = 1.16, P<0.05, respectively). Irrespective of the different larval density tested, we observed an overall mean (±SE) number of pupae which did not pass through the sieve equal to 6247 (971) with a percentage of male of 54.36% (6.56).

### Experiment 2. Effect of brewer's yeast on *Ae. albopictus* pupal production and sex separation efficacy

In the first preliminary test (EXP 2A), statistical difference was observed in the number of pupae passed through the sieve at 24 h from pupation onset (NPP) when reared on the IAEA-BY diet at different larval densities (F_(1,8)_ = 29.93, P<0.001; Table 2, EXP 2A). The percentage of males observed in the pupae sieved (%MPP) did not vary with the larval density tested (F_(1,8)_ = 0.59, P>0.05). At the larval density of 2 larvae/ml, the pupal production (NPP/NL_1_) was statistically lower in comparison with rearing conducted at 3 larvae/ml (F_(1,8)_ = 5.66, P<0.05; Table 2, EXP 2A). In the second experiment (EXP 2B), conducted at a density of 3 larvae/ml, the number of pupae produced at 24 h from pupation onset (NP) did not differ significantly with larval diet (F_(1,4)_ = 4.41, P>0.05; Table 2, EXP 2B). When mechanically sexed, the pupae collected at 24 h from pupation onset in the two diet treatments showed no statistical difference in the number of pupae that passed through the sieves (NPP) (F_(1,4)_ = 0.02, P>0.05; Table 2, EXP 2B) while the percentage of males observed in the pupae that passed through the sieves (%MPP) varied significantly according to the larval diet (F_(1,4)_ = 34.23, P<0.01) showing higher values when larvae were fed on IAEA-BY. The pupal production (NPP/NL_1_) did not differ significantly according to larval diet treatments (F_(1,4)_ = 0.03, P>0.05; Table 2, EXP 2B).

### Experiment 3. Effect of water temperature on *Ae. albopictus* pupal production and sex separation efficacy

Larvae reared at water temperatures of 25.5°C and 26.5°C pupated one day later (day 6 from initial L_1_ introduction) than larvae reared at temperatures of 28.5°C and 29°C (5 days from L_1_ introduction). As reported in Table 2 (EXP 3), the number of pupae passing through the sieves (F_(3,34)_ = 5.93, P<0.01), the percentage of males observed (F_(3,34)_ = 4.16; P<0.05) and the pupal production values (F_(3,34)_ = 4.34, P<0.05) vary significantly with water temperature.

### Experiment 4. Effect of larval trays settings on *Ae. albopictus* pupal production and sex separation efficacy

The number of pupae collected after mechanical sex separation (F_(1,31)_ = 0.02, P>0.05), their percentage of males (F_(1,31)_ = 2.07, P>0.05) and the pupal production (F_(1,31)_ = 0.29, P>0.05) did not differ significantly according to the larval rearing setting used (Table 2, EXP 4).

### Experiment 5. Effect of water temperature on *Ae. albopictus* pupal production and sex separation efficacy using the rack

The number of pupae passing through the sieve and the pupal production did not vary significantly with water temperature (F_(1,7)_ = 0.71, P>0.05 and F_(1,7)_ = 0.90, P>0.05, respectively), while the percentage of male pupae was statistically different (F_(1,7)_ = 25.89, P<0.01; Table 2, EXP 5).

## Discussion

The larval rearing of *Ae. albopictus* in the new IAEA unit supported a high larval survival rate up to pupation irrespective of the larval density tested in the range of 2–4 larvae/ml, confirming the high adaptability of this species at different levels of competition and resource availability during larval stage [31], [32] with minimal impact on survivorship [33].

There was minimal evaporation of water from the trays during the experiments and no addition of water was necessary during any tests where trays were placed on shelves or inside the rack. The mean water temperature (±SD) measured in the isolated tray was 2.4 (±0.3)°C cooler than the ambient temperature in the climate-controlled room, whereas the water temperature in the trays stacked inside the rack was 3.5 (±0.2)°C lower than the ambient temperature. Evaporation levels and water temperature observations were consistent with previous observations [10].

The tests using the IAEA diet for larvae kept at a density of 2 and 3 larvae/ml resulted in high pupal production by 24 h after the onset of pupation. Rearing at a larval density of 4 larvae/ml resulted in the lowest production of pupae probably due to overcrowding and for this reason was not further investigated (NPP/NL_1_; Table 2, EXP 1). The comparison between the larval density of 2 and 3 larvae/ml using a diet supplemented with brewer's yeast (IAEA-BY) confirmed the advantage of using the higher larval density for the purpose of mass rearing.

The ability to produce large numbers of pupae within 24 h of pupation onset is fundamental for any *Aedes* mass rearing facility where mechanical sexing is relied upon. Because of protandry, *Aedes* male larvae tend to pupate before the females, therefore pupae collected within 24 h from the onset of pupation are mostly males. Mosquito larvae must achieve a critical weight before pupating and this critical weight is higher for female larvae than for male larvae [34]. Protandry can be exploited to facilitate the selection of male pupae in *Aedes* facilities and, as also demonstrated in this experiment, pupation occurring late, (between 48 and 72 h from pupation onset) is less productive in terms of overall collection of male pupae. Our results confirmed previous findings in *Ae. albopictus* [21].

Together with a high male pupae recovery at 24 h after the onset of pupation, it is also important to produce uniformly sized pupae in order to support a consistent and effective sexing procedure. The rate of male pupae obtained with the mechanical sexing procedure was in the range of 10–13% of the initial number of L_1_ (or 20–26% of the total retrievable males). In view of the high percentage of males observed in the pupae collected at 24 h from onset of pupation before the mechanical separation (%M 24 h; Table 1), consideration should be given to derive more effective techniques for sex separation.

Although the size difference between male and female pupae resulting from rearing at different larval densities were fairly constant, certain variability can create an overlap of sizes [21] thereby reducing the efficiency of sexing methods based on pupal dimorphism [35]. Nevertheless the mechanical separation efficacy could perhaps be improved by changing the shape of the sieve mesh according to the shape of the cephalothorax of the pupae [29] or formed by long slots as proposed by Sharma [36]. Using a smaller number of pupae per separation batch may also help improve the efficacy of the separation method ensuring greater space for each pupa thus reducing the possible disturbance created by pupal contacts during attempts to pass through the sieve. Repeated sexing procedures with different mesh sizes or different mechanical methods [36], [37], [38], [39] in sequence could increase the recovery rate of male pupae (currently ca. 25% of total male produced) even if higher levels of stress would inevitably be caused to the pupae being processed. To improve male separation, the behavioral differences at adult stage could also be exploited by offering blood meals spiked with insecticide or toxicants [35], [40], [41] or investigating the possibility to extract males from the conspecific population in cages by generating specific female sound emissions [42]. Finally the possibility to enhance the sexual dimorphism and the protandry at pupal stage either through the effect of specific larval diet components or by the selection of particular strains obtained with artificial disassortative mating is under ongoing investigations (Bellini unpublished data).

While a maximum value of 1% female contamination would be acceptable for releases of sterile male mosquitoes in areas free from endemic transmission of arboviruses [43], this value needs to be strongly reduced for areas with endemic and recurrent epidemic events. The different larval densities tested did not alter the percentage of male pupae collected from the trays at each collection time after pupation onset. Even after sexing procedures, the percentage of males in the batches of pupae that passed or did not pass through the sieves was not affected by the larval rearing density. However, none of the larval rearing densities tested with the IAEA diet gave satisfactory results in terms of the percentage of males separated at 24 h from pupation onset. With the use of the diet enriched with brewer's yeast (IAEA BY), a significant improvement in the accuracy of the sex separation was observed. This specific diet component could possibly increase the dimensional difference between sexes at pupal stage contributing to a better male selectivity of the sexing method. The brewer's yeast seems to be a good supplementary component for the IAEA diet for the rearing of *Aedes* species, even if further investigation is required to assess the relationship between diet composition, sex separation efficiency and reliability, and adult fitness.

Larval rearing conducted at water temperatures of 25.5°C and 26.5°C resulted in a higher number of pupae produced probably due to the increased larval development time at these two temperatures. However, in these rearing conditions, an unacceptable residual presence of females after the mechanical sex separation was observed. Furthermore, considerable cost reduction in terms of space, labor, and larval diet requirements can be expected if overall rearing duration is reduced.

Rearing mosquito larvae in trays stacked inside the rack resulted in reduced efficacy of the sexing procedure when the water temperature was set at 29°C. This rather high water temperature apparently induced considerable acceleration in the larval development time, thus reducing the possibility to exploit the natural male pupal precociousness typical of this species (protandry). A water temperature of 28°C seems to be the optimal temperature using the IAEA/FAO larval rearing unit.

The larval development time did not differ when rearing in individually placed trays, or in trays stacked within the dedicated rack. The results obtained from the rearing in the rack setting confirmed that with a water temperature of 28°C, the pupal production at 24 h from first pupation and the sex separation efficiency were consistent with previous studies on *Ae. albopictus* [44] and *Ae. aegypti* [45].

**Figure 1:**
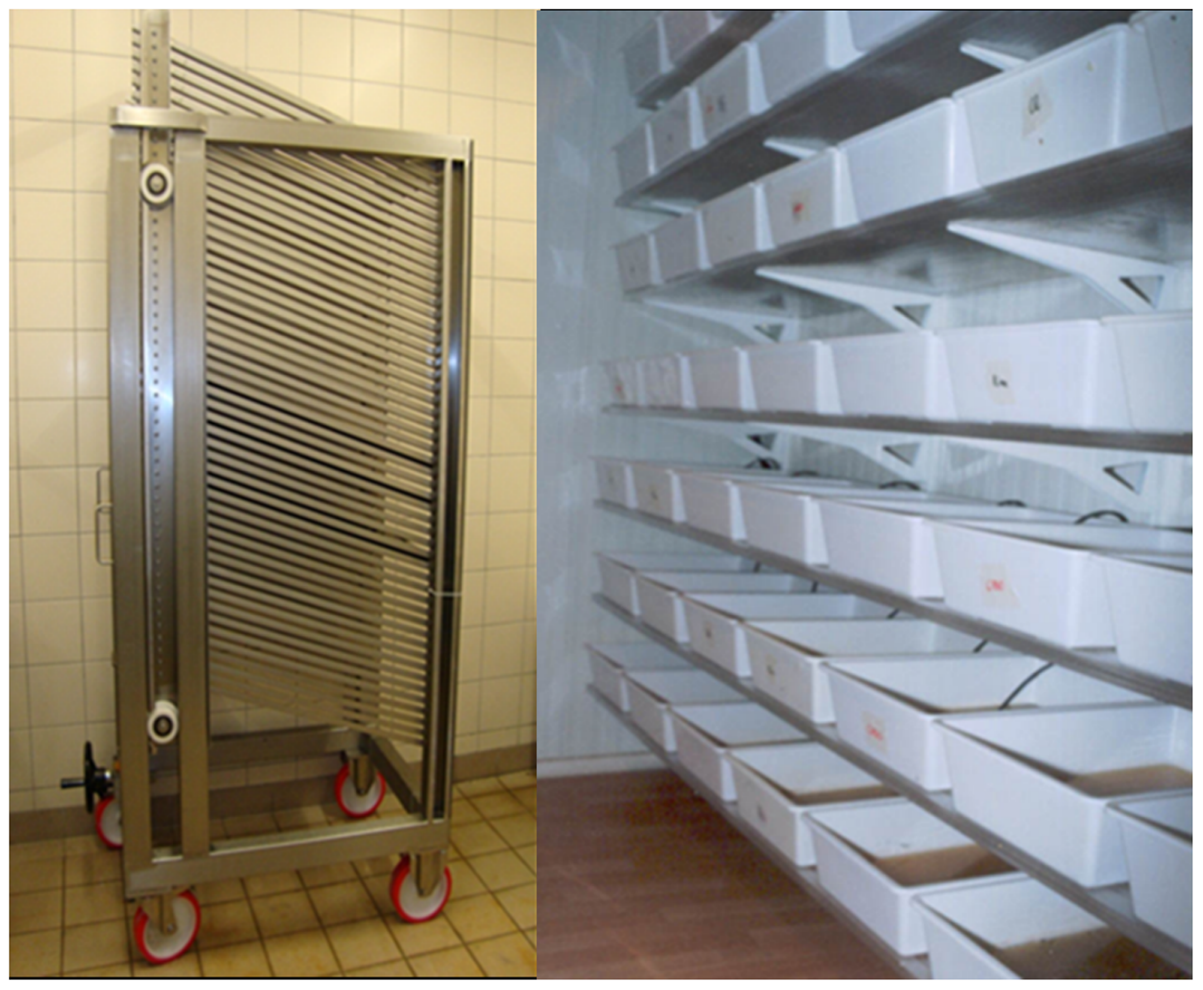
FAO/IAEA larval rearing unit (left) and standard laboratory trays used for *Aedes albopictus* larval mass rearing procedures (right).

With the use of this larval rearing unit, an average production of 100,000 male pupae per week can be achieved in about 2 sqm of laboratory space. This shows a considerable reduction in space requirements when compared to the rearing method previously exercised [13] [21], where the same pupal production required the use of ca. 200 plastic trays which occupied two climate-controlled rooms of 5 square meters each (Fig. 1).
